# Nonequilibrium phase transition of a one dimensional system reaches the absorbing state by two different ways

**DOI:** 10.1038/s41598-023-48394-w

**Published:** 2023-12-06

**Authors:** M. Ali Saif

**Affiliations:** Department of Physics, University of Amran, Amran, Yemen

**Keywords:** Mathematics and computing, Information theory and computation, Statistical physics, thermodynamics and nonlinear dynamics

## Abstract

We study the nonequilibrium phase transitions from the absorbing phase to the active phase for the model of diseases spreading (Susceptible-Infected-Refractory-Susceptible (SIRS)) on a regular one-dimensional lattice. In this model, particles of three species (S, I, and R) on a lattice react as follows: $S+I\rightarrow 2I$ with probability $\lambda$, $I\rightarrow R$ after infection time $\tau _I$ and $R\rightarrow I$ after recovery time $\tau _R$. In the case of $\tau _R>\tau _I$, this model has been found to have two critical thresholds separating the active phase from absorbing phases. The first critical threshold $\lambda _{c1}$ corresponds to a low infection probability and the second critical threshold $\lambda _{c2}$ corresponds to a high infection probability. At the first critical threshold $\lambda _{c1}$, our Monte Carlo simulations of this model suggest the phase transition to be of directed percolation class (DP). However, at the second critical threshold $\lambda _{c2}$ we observe that the system becomes so sensitive to initial values conditions which suggest the phase transition to be a discontinuous transition. We confirm this result using order parameter quasistationary probability distribution and finite-size analysis for this model at $\lambda _{c2}$.

## Introduction

The nonequilibrium phase transition from the active states to the absorbing states has attracted a lot of scientific community efforts recently^1–3^. One of the most important efforts in this field is concerned with classifying nonequilibrium systems into universal classes. In this sense, the directed percolation class (DP) is the most important in the nonequilibrium phase transition to the absorbing states. Many models belong to this class, for example, a contact process (CP), Domany-Kinzel cellular automaton (DK), Ziff-Gulari-Barshad (ZGB) model, pair-contact process (PCP), threshold transfer process (TTP) and branching annihilating walks with an odd number of offspring^4–10^. According to Janssen-Grassberger criterion^2,3,11^ a model should belong to the DP universality class if the model satisfies the following conditions, displays a continuous transition into a unique absorbing state with a positive one-component order parameter, with short-range interactions and without quenched disorder or additional symmetries. DP class seems to be even more general, systems belong to this universality class even if they violate some of the previous criteria. For example in the long-range interactions^12^ or certain models with many absorbing states^13–16^ or fluctuating passive states^17^.

Another important class of the nonequilibrium phase transitions to the absorbing state is the voter universality class. This class has been observed in special cases of models with a two symmetric ($Z_2$ symmetry) absorbing state^3,18,19^. Models such as compact directed percolation (CDP), the $2A\rightarrow \phi$ and the $2A\rightarrow A$, the cellular automaton version of the nonequilibrium kinetic Ising model and Lévy-flight anomalous diffusion in annihilating random walks belong to this class. Parity-Conserving universality class (PC)^3,18,19^ is another universality class to absorbing state. This class characterizes those models which conserve the number of particles modulo 2. Examples are the one-dimensional kinetic Ising models which combined finite temperature spin-exchange dynamics and zero temperature spin-flip^20^, branching and annihilating random walks with an even number of offspring^21^ and parity-conserving class of surface-catalytic models^22^.

Nonequilibrium discontinuous phase transitions from an active state to an absorbing state have been also observed in the dimensions higher than one in many cases. For example in a two-dimensional ZGB model and its modifications^4,6,7,23–27^, a two-dimensional reaction-diffusion contact-process-like model^28–30^, two lattice versions of the second Schl$\ddot{o}$gl model (SSM)^31,32^, a two-dimensional naming game model^33–35^, two and four dimensions deterministic conserved threshold transfer process^36,37^ and the prisoner’s dilemma with semi-synchronous updates^38^ on two dimensions. However, discontinuous phase transition to absorbing states has been rarely seen in one dimension. This is because the fluctuations in low dimensions are strong which makes the continuous phase transitions likely to occur. Hinrichsen^1,2^ argued that first-order phase transition can not occur in one-dimensional nonequilibrium systems unless there are extra symmetries, conservation laws, long-range interactions, or special boundary conditions. By any means in one dimension, the first order phase transition has been observed in systems with conserved density^37,39^, models with long-range interactions^40^ and in the systems with multi-component^41,42^. For a two-species reaction-diffusion process on one dimension the renormalization group methods predict a first-order phase transition^43,44^, however, the numerical simulations of that model have yielded results in disagreement with the renormalization group prediction^45,46^. The model candidate to violate Hinrichsen’s argumentations is the triplet creation model (TCM). This model is a single component and does not possess a conservation law or long-range interactions. A preceding study by Dickman and Tomé^30^ had suggested the first order phase transition of this model for a high value of diffusion rate ($D\ge 0.9$). In sequence, Cardoso and Fontanari modified that value to be $D\ge 0.95$^47^. Recently, the simulation results of the TCM model by Park^48^ have shown that the phase transition of this model is continuous for any value of $D\le 0.98$. A more recent study of this model by Ódor and Dickman^49^ suggests a continuous phase transition for any value of $D<1$.

In this work, we are going to study the phase transition from the absorbing state to the active state of the epidemics spreading model SIRS (Susceptible- Infected- Refractory- Susceptible) on a one-dimensional regular network. This model has been proven to own a two critical threshold^50^. We are interested in studying the phase transition close to those critical thresholds. This work is organized as follows. In Sect. 2, the model and simulation methods are described. Simulation results close to the first critical point of this model are presented and discussed in Sect.  3. Simulation results close to the second critical point of this model are given and discussed in Sect. 4. Conclusions are given in Sect. 5.

## Model and methods

The model of epidemics spreading SIRS on the networks can be described as follows^51^: consider a population of *N* particles residing on the sites of a lattice in which each particle is connected to *k* of its nearest neighbors. The particles can exist in one stage of three stages, susceptible (*S*), infected (*I*), and refractory (*R*). The interaction between the particles on the lattice is as follows: the particles in state *I* on the network can infect any one of their neighbors which are in state *S* with probability $\lambda$ at each time step ($S+I\rightarrow 2I$). The particles in state *I* pass to the *R* state after an infection time $\tau _I$ ($I\rightarrow R$). The particles in state *R* return to the *S* state after a recovery time $\tau _R$ ($R\rightarrow I$). During the *R* phase, the particles are immune and do not infect.

**Figure 1:**
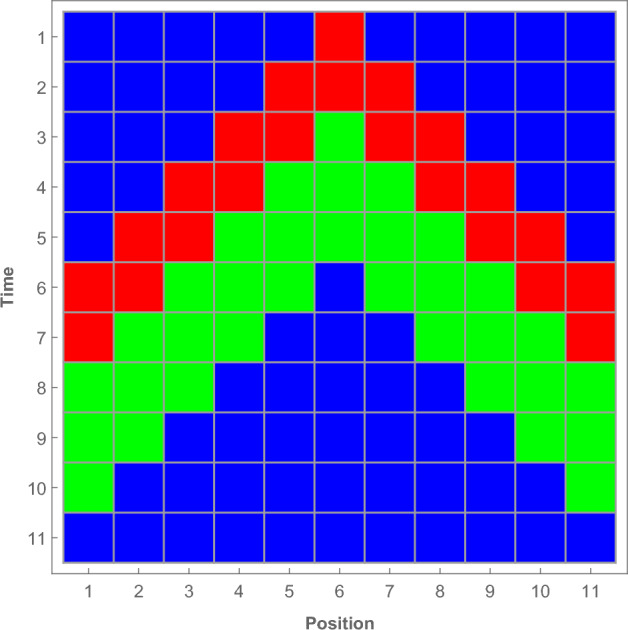
Space-time evolution of lattice of 11 sites with periodic boundary condition when $\lambda =1$, $\tau _I=2$, $\tau _R=3$ and $k=1$ (Blue: *S*, Red: *I* and Green: *R*).

For this model on the networks as it has been proven in Ref.^50^, we have to distinguish between the following two cases: The first case happens when $\tau _I\ge \tau _R$, in this case SIRS model owns only a one critical threshold $\lambda _{c}$ separates the active phase from absorbing phase. The second case happens when $\tau _I\le \tau _R$, in this case, the SIRS model will own two critical thresholds $\lambda _{c1}$ and $\lambda _{c2}$, in which the system is active in between them and dies outside of them. In the second case, the first critical threshold $\lambda _{c1}$ corresponds to the situation where the infection probability $\lambda$ is low. So in this case, the spreading of infection is limited and local therefore, and when $\lambda <\lambda _{c1}$, the system will evolve to the absorbing state where all particles become susceptible (state *S*). In contrast the second critical threshold $\lambda _{c2}$ corresponds to the situation where the infection probability $\lambda$ is high. Therefore, in this case, the infection will spread globally and quickly throughout the entire network. Now let us ask this question: What will happen for the system when $\lambda$ is high enough such that, all the particles in the network become infected during a time that is less than or equal to $\tau _I$? In this case, the maximum value for the infection time difference between any two connected particles in the network will not be greater than $\tau _I$. Where $\tau _R>\tau _I$, so during the time which is not longer than $\tau _I$ all the particles will approach the state *R* in which the network becomes free of infected particles (active particles)^50^. Therefore the state where all the particles become infected in the network is also an absorbing state for this model. However this absorbing state is un-stationary where the particles stay in this state only for a time which is not longer than $\tau _I$ before they cross to the state *R* and then they settle down in the stable absorbing state *S* (see Fig. 1). So we can say that, when $\tau _I\le \tau _R$ the SIRS model has two absorbing states, even the second absorbing state is not stable but if the system reaches it, then the system will evolve surely to the stable absorbing state (first absorbing state *S*).

As aforementioned, the absorbing state of this model is the state where the lattice becomes free of infected particles, i. e. the state *S*. The SIRS model approaches this absorbing state in two different ways. At $\lambda _{c1}$ the model reaches an absorbing state due to that, the strength of infection is very low hence, the average number of susceptible particles infected by an already infected one during the time $\tau _I$ is less than one. Whereas at $\lambda _{c2}$ the strength of infection is high such that, each infected particle infects all of its neighbors during the time $\tau _I$. The second critical threshold is equivalent to the state where all particles reach the state *I* during a time that is less than or equal to $\tau _I$. In this case where $\tau _R>\tau _I$ then, instantaneously all the particles will approach the state *R* followed by the absorbing state *S* during a time which is not longer than $\tau _R$. we can consider the state where all particles on the lattice are infected (state *I*) as an absorbing state of this model however, this state is un-stationary and will end up in the absorbing state *S*. In Fig. 1 we show the space-time evolution for a one-dimensional lattice of 11 sites with periodic boundary conditions. In this lattice, each particle is connected to its first two neighbors. We set the infection probability $\lambda =1$, infection time $\tau _I=2$, recovery time $\tau _R=3$ and the infection starts with one particle on the center of the lattice. It is clear from the figure that, all particles on lattice will approach the absorbing state *S* after 11 time steps.

We simulate this model on a regular one-dimensional lattice with periodic boundary conditions in which each particle on the lattice is connected to $k=3$ of its nearest neighbors on each side. The system updates synchronically. In this work, we fix the values of the infection time and recovery time to be $\tau _I=7$ and $\tau _R=9$ unless we state differently. The order parameter $\rho (t)$ is the density of infective particles *I* (active particles)

1 $$\begin{aligned} \rho (t)=\frac{\left\langle \sum _j I_j(t)\right\rangle }{N} \end{aligned}$$

where *N* is the total number of lattice sites and $\left\langle ...\right\rangle$ stands for the average over ensembles. Steady state of the order parameter $\rho _s$ is the state when $\rho _s \equiv {{\mathop {t\rightarrow \infty }\limits ^{lim}}}\rho (t)$.

**Figure 2:**
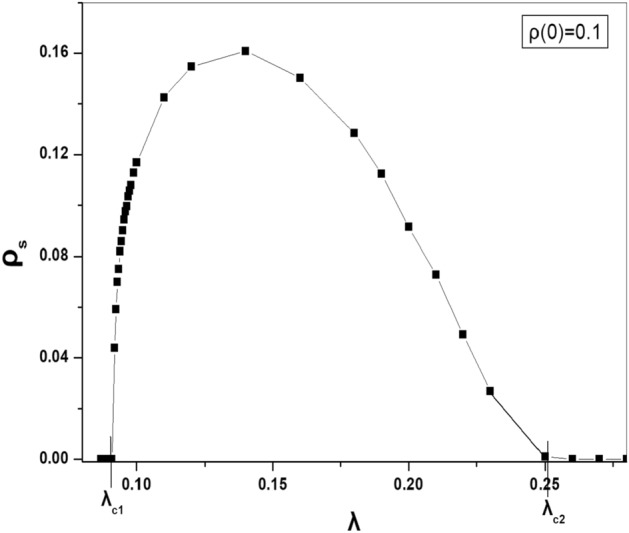
The steady state of the density of particles $\rho _s$ at various values of the $\lambda$ when $N=10^4$, $k=3$, $\tau _I=7$ and $\tau _R=9$.

In Fig. 2, we recreate the steady state of the density of active particles as a function of $\lambda$ as it was given in Ref.^50^. For each point in Fig. 2, we start the simulations from the initial density of active particles $\rho (0)=0.1$. We take the average over 100 configurations after discarding $10^4$ initial time steps. Figure 2 shows clearly the two critical thresholds and we are interested to study the phase transition at them.

## Phase translation at the first critical point $\lambda _{c1}$

**Figure 3:**
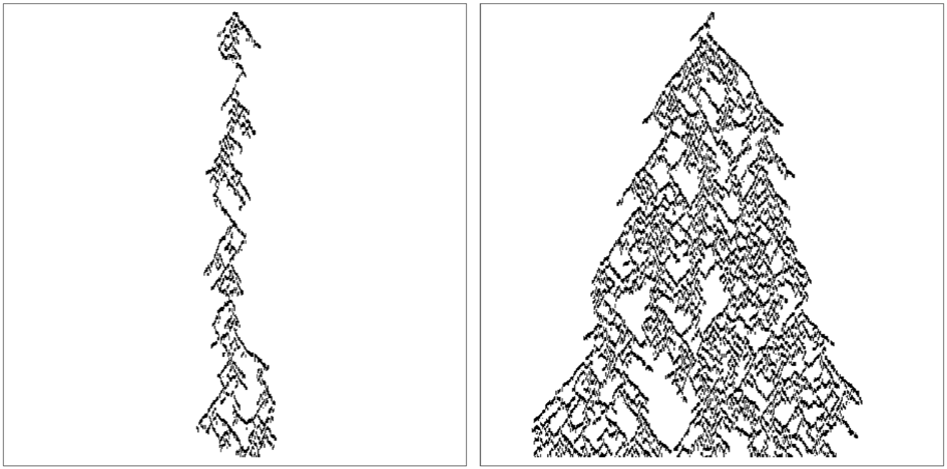
Typical space-time evolutions for $\lambda =0.090$ (right) and $\lambda =0.094$ (left). Simulation starts from a single active particle (black) and time increases downward.

For a general view of the kind of phase transition at the first critical point, we start the simulation of this model with the typical space-time evolution beside $\lambda _{c1}$. In Fig. 3 we show the typical space-time evolution of this model when the simulation starts initially from a single active seed located at the center of the lattice for the values of the parameters $\lambda =0.090$ and $\lambda =0.094$. Figure 3 seems to be similar to the typical space-time evolution of the systems that undergo DP phase transition^2^.

**Figure 4:**
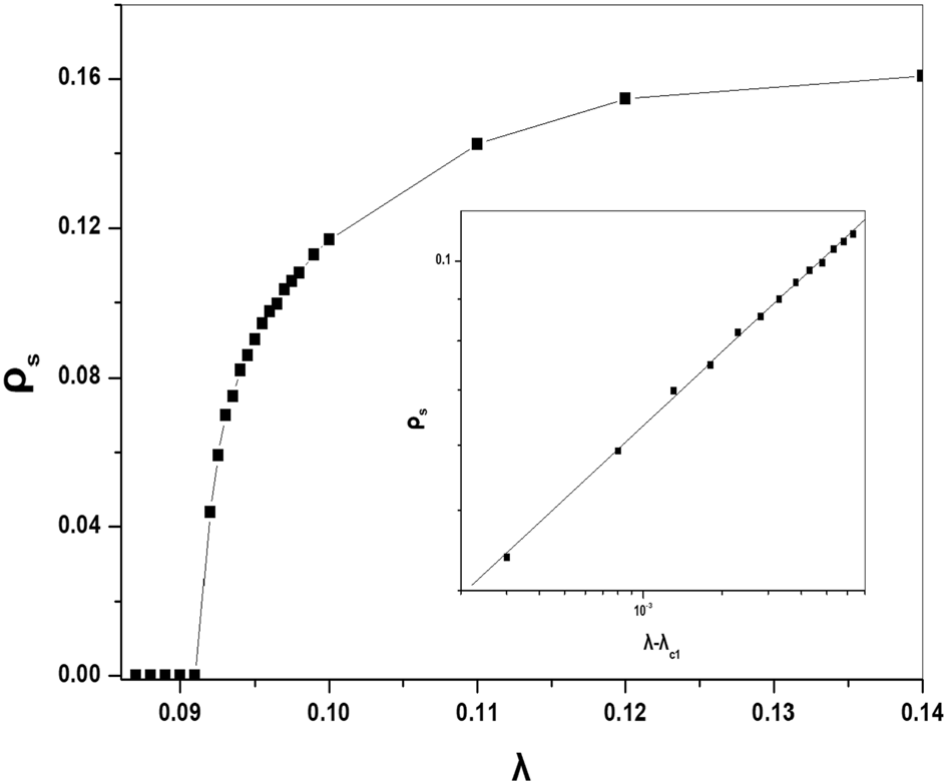
The steady state of the density of particles at various values of the $\lambda$ for the same parameters in Fig. 2. The inset is a Log-log plot of the $\rho _s$ as a function of the distance to the critical point.

To confirm if the phase transition, in this case, is in the DP universality class, we are going to calculate some values of the critical exponents of this model, and before that, we will determine the value of critical point $\lambda _{c1}$. In Fig. 4 we plot the average value of the density of active particles $\rho _s$ at various values of the infection probability $\lambda$. In the simulation, we use a lattice of size $N=10^4$ averaged over 200 realizations after discarding the first $10^4$ time steps. Figure 4 shows clearly that, the system crosses from the absorbing phase to the active phase at a specific value of the parameter $\lambda$. For the best estimations, the value of the critical point seems to converge to $\lambda _{c1}=0.906\pm 0.004$. Using this result we can determine one of the critical exponents related to this model where, it is known that, for the continuous phase transitions and as the control parameter $\lambda$ approaches the critical point $\lambda _c$, the stationary value of the order parameter $\rho _s$ vanishes asymptotically according to a power law as follows^1–3^:

2 $$\begin{aligned} \rho _s\sim (\lambda -\lambda _c)^{\beta } \end{aligned}$$

The Inset of Fig. 4 shows the logarithmic plot of $\rho _s$ as a function of the distance from the critical point $(\lambda -\lambda _{c1})$, which shows clearly the power law behavior. The estimated value of the critical exponent $\beta$ from the inset of Fig. 4 gives us $\beta =0.281\pm 0.005$ which consists very well with the value of $\beta =0.276$ for the $(1+1)$ DP universality class^1–3^.

To extract a further critical exponent, we perform time-dependent Monte Carlo simulations of this model starting from a fully occupied lattice. As we know for continuous phase transitions, at the critical point $\lambda _c$, the time evolution of the order parameter $\rho (t)$ decays asymptotically according to the following power law^1–3^

3 $$\begin{aligned} \rho (t)\sim t^{-\delta } \end{aligned}$$

Where $\delta$ is the critical exponent which equal to 0.159464(6) for DP universality class in the $1+1$ dimension^2^.

**Figure 5:**
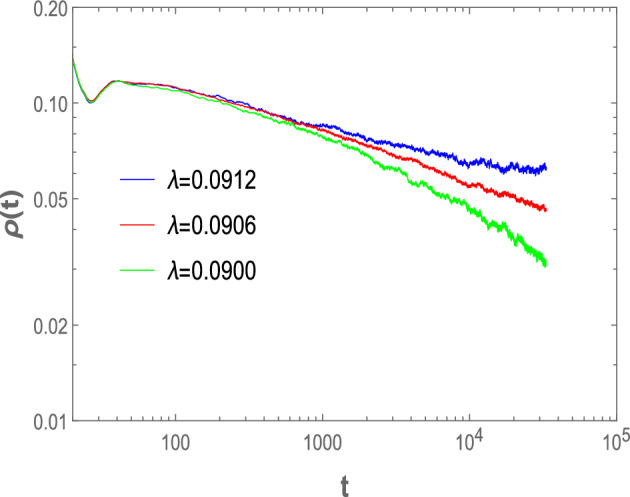
The density of active particles as a function of $\lambda$ when $N=10^4$, $k=3$, $\tau _I=7$ and $\tau _R=9$.

Figure 5 shows the density of active particles $\rho (t)$ as a function of time on a logarithmic scale. At the critical point, the system clearly shows a power law decay of the active particles. For the best fitting, the value of the critical exponent we find to be $\delta =0.159\pm 0.005$ which is again consistent with the value of the critical exponent for the DP universality class in the $1+1$ dimension.

Hence we can confirm that, for the case when $\tau _I<\tau _R$ the phase transition from the absorbing state to the active state for the SIRS model at the first critical point $\lambda _{c1}$ is of kind DP universality class in $(1+1)$ dimension. Here we should mention to that, close to $\lambda _{c1}$, the values of $\lambda$ are low enough in which the stable absorbing state *S* is dominated state on the system i. e., it is impossible for the system to approach the un-stationary absorbing state *I* in this case. Therefore the accessible absorbing state for the system close to the first critical point is only the state *S*, consequently in this case the system satisfies the Janssen Grassberger criterion except that this model is a multi-component system.

## Phase transition at the second critical point $\lambda _{c2}$

**Figure 6:**
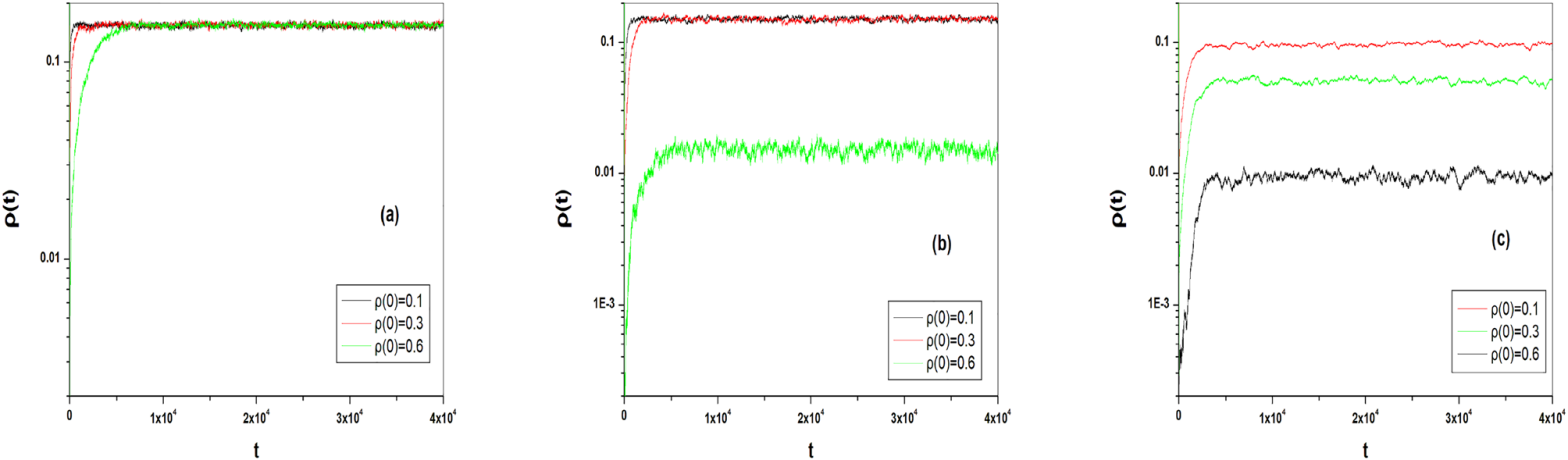
The time evolution of the average value of density of particles as a function of time at different values of initial conditions for $N=10^4$, $k=3$, $\tau _I=7$ and $\tau _R=9$: a) for $\lambda =0.12$ b) for $\lambda =0.16$ c) for $\lambda =0.20$. For each curve, we averaged over 200 realizations.

As we increase the value of $\lambda$ toward the second critical point $\lambda _{c2}$ we observe that at a specific value of $\lambda$ (which is $\lambda >0.15$ for the system of size $N=10^4$ other parameters are same to the parameters we have used in the previous section) the steady state of the average density of infected particles $\rho (t)$ becomes strongly dependent on its initial value of density of infected particles $\rho (0)$. To manifest this fact, Fig. 6a–c show the average value of $\rho (t)$ as function of time when the values of $\lambda$ are $\lambda =0.12$, $\lambda =0.16$ and $\lambda =0.20$ respectively. Figure 6b shows three curves of $\rho (t)$ as a function of time in which we start the simulations from different initial values of $\rho (0)$. This figure shows clearly that, when the simulation starts with the initial value $\rho (0)=0.1$ or $\rho (0)=0.3$, the density of active particles $\rho (t)$ attains the same value. However if the simulation starts with $\rho (0)=0.6$, the steady state of $\rho (t)$ approaches a different value. As the value of $\lambda$ increased in Fig. 6c the situation becomes more apparent, where of each value of the initial conditions $\rho (0)=0.1$, $\rho (0)=0.3$ or $\rho (0)=0.6$ the steady state of $\rho (t)$ reaches to different values. Figure 6a is given for comparison where in this case the value of $\lambda$ is low in which the steady state of $\rho (t)$ approaches the same value for each value of initial conditions $\rho (0)$. In this case, the system does not show dependence on its initial conditions. Here we should mention that, in Monte Carlo simulations of Fig. 6, we take the average of $\rho (t)$ over all configurations those survive or not. Another point we mention here is that the sensitivity of the steady state of the order parameter to initial conditions in the model of disease spreading has been also observed in the minimal vaccination-epidemic model^52^.

**Figure 7:**
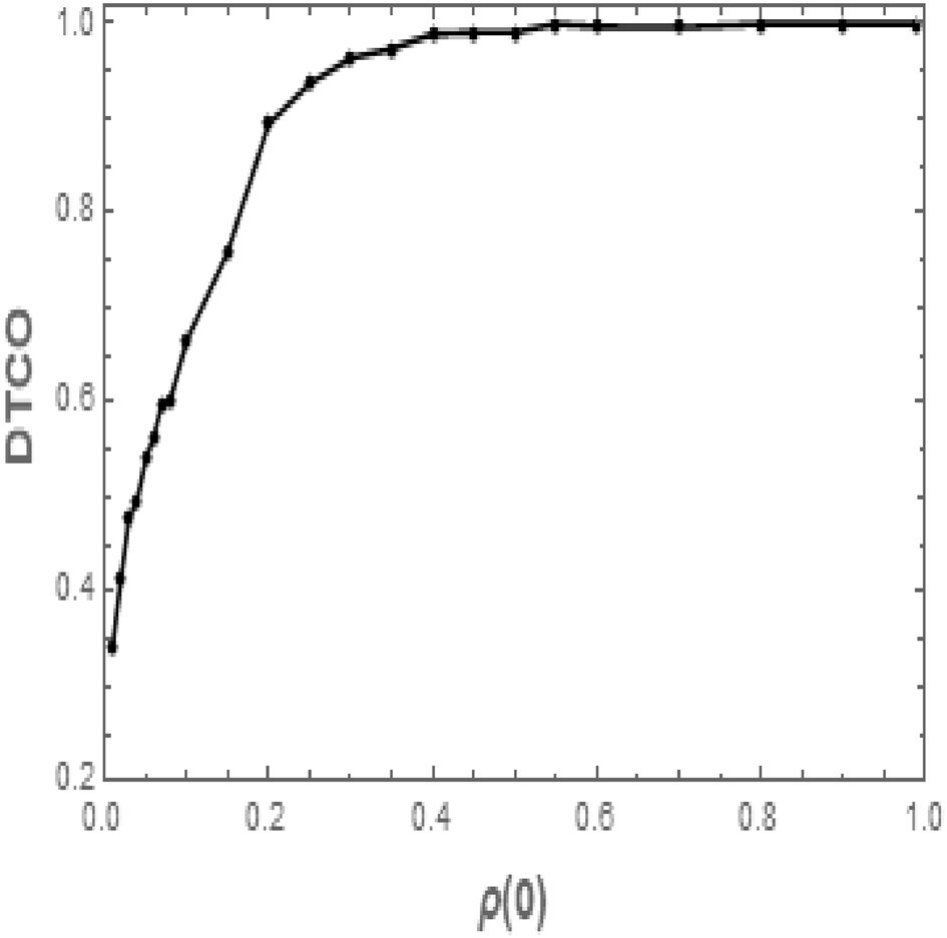
The density of trapped configurations (DTCO) as a function of time at different values of initial values conditions when $N=10^4$, $k=3$, $\tau _I=7$, $\tau _R=9$, and $\lambda =0.16$, each point in the figure averaged over 2000 configurations.

With careful inspection for the time evolution $\rho (t)$ of this system when the value of $\lambda$ is $\lambda >0.15$, we find that taking the average of $\rho (t)$ over all configurations is the reason behind the difference in the steady state of $\rho (t)$ as the initial values $\rho (0)$ are changed. Whenever some configurations reach so quickly to the absorbing state other configurations survive for a long time. Here we will consider the configurations that approach the absorbing state during a time that is not longer than $\tau _I+\tau _R$ (in our case that time is 17 time steps) as trapped configurations. Incidentally, this time on average is exactly the time that the particles need to go through one infection cycle. Additionally, we observe that for any fixed values of $\rho (0)$ the density of trapped configurations increases as the value of $\lambda$ becomes higher. In contrast for fixed values of $\lambda$, increasing the value of $\rho (0)$ causes increasing in the density of trapped configurations. The relation between the density of trapped configurations (DTCO) and initial active particles $\rho (0)$ when the value of $\lambda$ is $\lambda =0.16$ is given in Fig. 7. In this figure, the trapped configurations are those configurations reach the absorbing state during the time which is less than or equal 17 time steps. Figure 7 shows clearly that, whenever the initial density $\rho (0)$ of active particles is $\rho (0)>0.4$, all the configurations are trapped configurations. However, for the values of $\rho (0)<0.4$, we can see an increase in the number of surviving configurations as the value of $\rho (0)$ becomes lower.

We can deduce from the previous results that, the chance for the system to approach the state where all the particles in the network are infected (absorbing state *I*) becomes possible as the value of $\lambda$ goes toward higher values. This possibility is enhanced as the density of initial values is increased. We can understand the relation between the values of infection probability $\lambda$ and the initial values of density of infected particles $\rho (0)$ for this model from the mean-field approximation (see Ref.^53^). Whereas the increase in the number of *I* particles at the next time will be proportional to the number of particles of kind *I* and *S* at this time, i. e. $I(t+1)\propto I(t)+\lambda S(t) I(t)$, then, if the value of $\lambda S(I) I(t)$ becomes high enough such that $I(t+1)=N$ the system will reach the un-stationary absorbing state *I*. Therefore, we can say that, for high values of $\lambda$ un-stationary absorbing state becomes the dominating state in the system.

**Figure 8:**
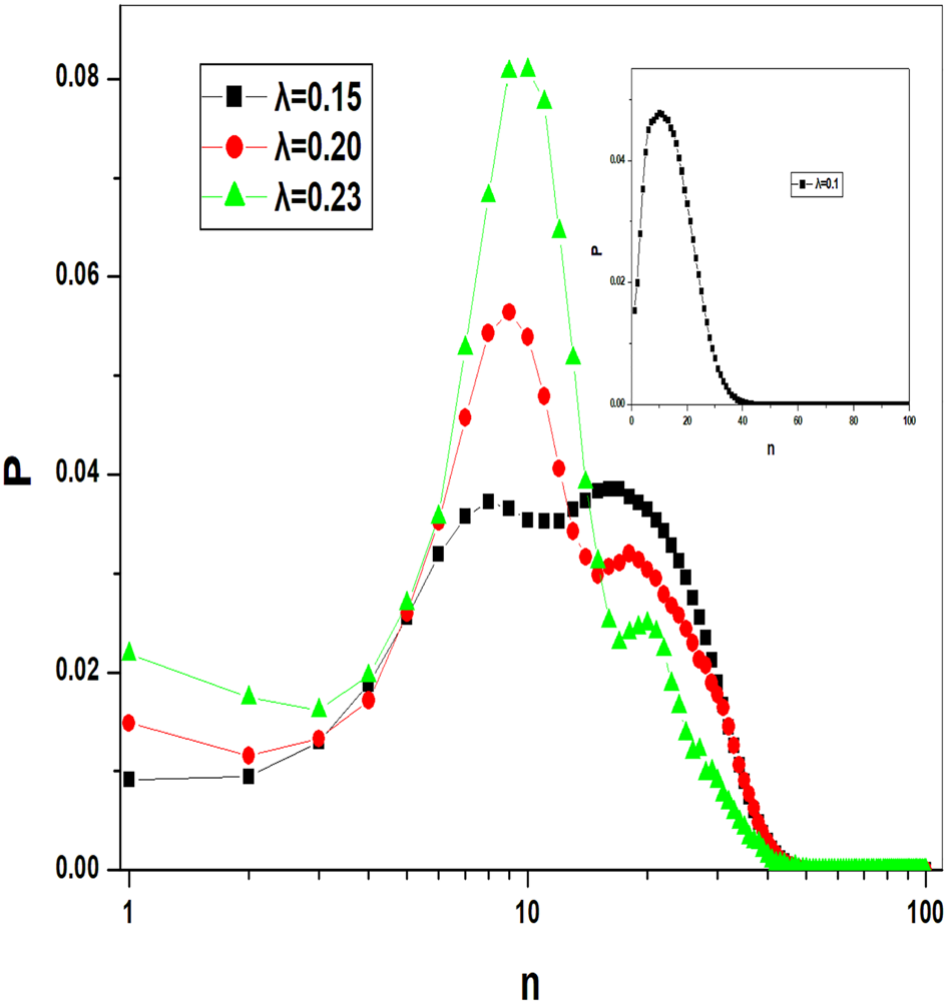
Cell-occupancy histogram distribution (P) for cells of 100 sites, when $\lambda =0.15$, $\lambda =0.20$ and $\lambda =0.23$, $N=10^3$, $k=3$, $\tau _I=7$ and $\tau _R=9$. Inset: Cell-occupancy histogram distribution for the same parameters except for $\lambda =0.1$.

Because of that dependence for the system on its initial values conditions near $\lambda _{c2}$, we have faced difficulty in determining the kind of phase transition or even accurately determining the critical point close to $\lambda _{c2}$. We should mention also that, we could not get any kind of power law behavior near the expected value of the critical point using the time-dependent dynamics starting from a fully occupied lattice or form a single active seed. However, according to Refs.^45,54^, the dependence of the initial values is an indicator of a discontinuous phase transition. Therefore, for a better understanding of the mechanism of the phase transition in this case, we perform the order parameter quasistationary probability distribution for this model. As claimed by Refs.^26,28,46,55–57^ the order parameter quasistationary probability distribution is bimodal in the neighborhood of a discontinuous phase transition in contrast to a continuous phase transition where there will be only a single pick. Figure 8 shows the results of our Monte Carlo simulations for the cell-occupancy histogram distribution (P) of our model for cells of 100 sites at the center of a lattice of $N=10^3$ particles, at various values of $\lambda$. The variable *n* in Fig. 8 is the number of active particles. The quasistationary distribution is clearly bimodal. This result enhances the assumption of the discontinuous phase transition of this model at $\lambda _{c2}$. For comparison, the inset of Fig. 8 shows the cell-occupancy histogram distribution in the vicinity of $\lambda _{c1}$ ($\lambda =0.1$), where the system undergoes a continuous phase transition. It is clear in this case there is only a single pick.

**Figure 9:**
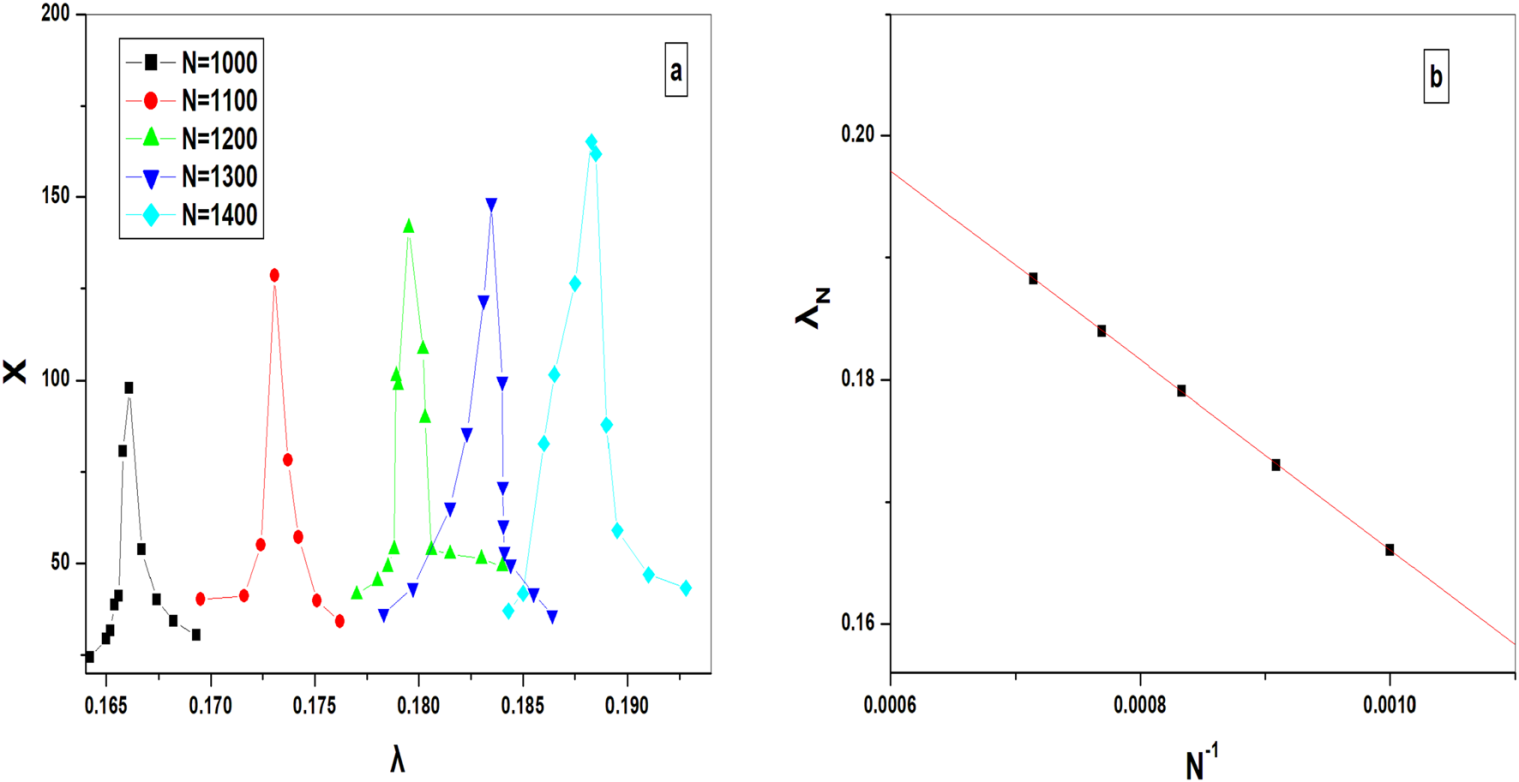
(**a**) Order parameter variance $\chi$ versus the parameter $\lambda$. (**b**) Value of $\lambda _N$ for which $\chi$ is maximum vs $N^{-1}$.

The results we find previously suggest strongly the phase transition at the second critical point $\lambda _{c2}$ to be discontinuous. However, for more clarification, we study here the quasistationary behavior of this system beside the critical point $\lambda _{c2}$. For that, we achieve a finite-size analysis, which is a more reliable procedure as recently proposed in Refs.^32,35^. According to this procedure, the difference between the pseudo transition point $\lambda _N$ (where *N* denotes the system volume) and the transition point $\lambda _{c2}$ scales with $N^{-1}$ according to the relation $\lambda _N=\lambda _{c2}+aN^{-1}$. Therefore, to determine accurately the value of $\lambda _N$ we use the system order parameter variance $\chi = N (\left\langle \rho ^2\right\rangle -\left\langle \rho \right\rangle ^2)$. This quantity has been proven to have a peak at the value of pseudotransition point $\lambda _N$^32,35^. We restrict our simulation here, only to surviving configurations. Figure 9a shows the order parameter variance $\chi$ as a function of $\lambda$. Whereas Fig. 9b shows how the values of pseudo transition points $\lambda _L$ scale with the values of system size $N^{-1}$. Extrapolation of $N\rightarrow \infty$ yields the critical point for this model to be $\lambda _{c2}=0.24\pm 0.01$.

**Figure 10:**
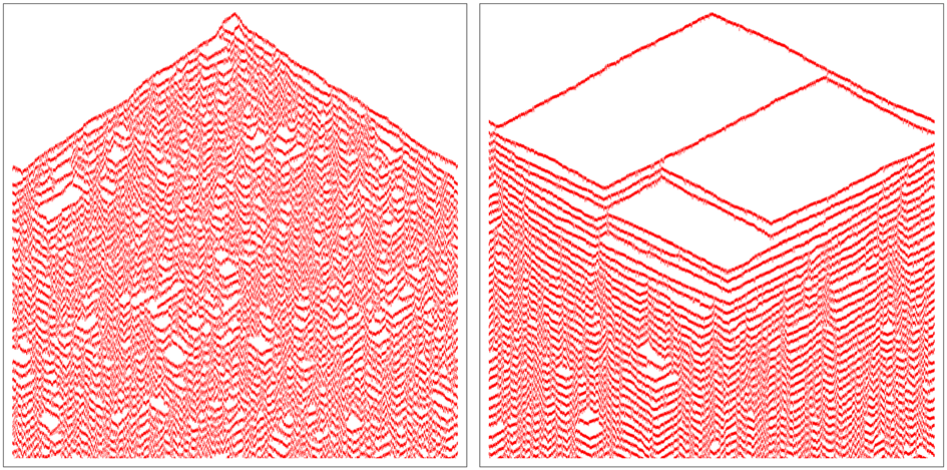
Space-time evolution of SIRS model from a single seed (red) when $\lambda =0.231$ (right) and $\lambda =0.241$ (left). Other parameters are $N=10^3$, $k=3$, $\tau _I=7$ and $\tau _R=9$.

The final point we have studied for this model is the space-time evolution of the active particles close to $\lambda _{c2}$. Figure 10 shows the time evolution of infected particles with red color during the time of $10^3$ time steps for a system of $N=10^3$ particles when the value of $\lambda =0.231$ and $\lambda =0.241$. In both figures, simulation starts at $t=0$ with all particles in the state *S* except for one active particle *I* at the center of the lattice. Figures clearly show that the spreading of active particles is compact in behavior reminds us the spreading behavior of CDP models. However, the difference here is that the particles have a finite time to stay in the active phase. The coexistence of small compact isolated islands of active particles with high regions of inactive ones is again indeed consistent with a discontinuous phase transition.

Finally, we mention that models of disease spreading such as a minimal vaccination-epidemic model and Susceptible- Infected-Susceptible (SIS) model have been also found to show either a continuous or a discontinuous active to absorbing phase transition^52,58,59^. Additionally, we can deduce some similarities between the phase transition in this model and the phase transition in the ZGB model on a two-dimensional lattice where both models have two critical thresholds. In both models the first critical threshold corresponds to the continuous DP class and the second critical threshold corresponds to the discontinuous phase transition. ZGB model has two absorbing states, the first one is at small values of adsorption rate (beside the first critical point) and the second one (beside the second critical point) is at high values of adsorption rate^4^. SIRS also has a one-absorbing state at a low infection rate (beside the first critical point) and an unstable absorbing state at a high infection rate (beside the second critical point).

## Conclusions

In summary, we have studied the phase transition from the absorbing phase to the active phase for the model of infection-spreading SIRS on the one-dimensional network. This model has been found to have two critical points where the infection survives in between those critical points and dies out outside of them. The two critical points correspond to a low infection rate and a high infection rate. Using Monte Carlo simulations we have found that, whereas the phase transition at the first critical point is of kind the DP universality class, the phase transition at the second critical point is of kind first order phase transition. In this manner, the presence of continuous and discontinuous phase transitions has been also confirmed in the models of disease spreading such as a minimal vaccination-epidemic model and SIS model^52,58,59^. We can also compare the phase transition in this model with the phase transition in the ZGB model. Both models have two critical points, the phase transition at the first critical point is of kind DP class and the phase transition at the second critical point is discontinuous. However, we should mention here that, the system we have studied here is one-dimensional whereas ZGB is a two-dimensional system (Supplementay Information Files).

### Supplementary Information

Supplementary Information 1.

Supplementary Information 2.

Supplementary Information 3.

Supplementary Information 4.

Supplementary Information 5.

Supplementary Information 6.

Supplementary Information 7.

Supplementary Information 8.

## Data Availability

All data generated or analysed during this study are included in this published article (and its Supplementary Information files).
